# Interaction of nanoplastics with metronidazole and ciprofloxacin: The *Trojan Horse* effect

**DOI:** 10.1371/journal.pone.0330708

**Published:** 2025-08-20

**Authors:** Emiliano Perez-Sanchez, Ana Martínez

**Affiliations:** Departamento de Materiales de Baja Dimensionalidad. Instituto de Investigaciones en Materiales, Universidad Nacional Autónoma de México, Coyoacan, Mexico City, Mexico; CSSRI: Central Soil Salinity Research Institute, INDIA

## Abstract

Microplastics and nanoplastics are everywhere, but little is known about their chemical reactivity. In this study we performed a Density Functional Theory study of polystyrene (PS, a common non-biodegradable thermoplastic polymer) and polylactic acid (PLA, a biodegradable polymer) to understand the capacity to react of these two nanoplastics. The chemical reactivity of these oligomers is investigated through their capacity to either donate or accept electrons and, therefore, their capacity to oxidize other molecules. To model nanoplastics, we used oligomers formed with different numbers of carbon atoms. PLA is a better electron acceptor than PS, which could be related to oxidation reactions. It has also been reported that the presence of micro- and nanoplastics in the environment increases the bioaccumulation of pharmaceuticals such as antibiotics. To investigate this idea, we calculated the interaction energies of PLA and PS oligomers with two antibiotics: ciprofloxacin and metronidazole. The results indicate that both can form stable compounds with these two antibiotics. This might be related to the *Trojan horse* effect, which refers to the idea that the presence of nanoplastics increases the bioaccumulation of drugs. These results contribute to understand the reactivity of these nanoplastics.

## Introduction

Polymers, or plastics, are fundamental in our society. They are everywhere and have multiple applications, from medicine to clothing. Plastic production is enormous, around 350 million tons per year. This production has generated more than 6.3 billion tons of polymer waste over time [1,2]. The presence of plastic waste distresses the ecosystems [3–5] as do the additives used during the polymerization process, which are released from discarded polymers and may induce detrimental effects on the well-being of animals and humans [6].

Plastics break into fragments due to mechanical processes and UV radiation. Long polymer chains break, giving rise to smaller chains [7]. The fragmentation of biodegradable plastics also happens through biodegradation. More fragments can be produced from biodegradable plastics than from non-biodegradable polymers. The fragmentation of plastics produces microplastics (smaller than 5 mm) and nanoplastics (smaller than 100 nm). Micro- and nanoplastics have become a topic of recent concern due their apparent ubiquity in the environment [8,9] and the limited knowledge about their chemical reactivity.

Microplastics have been associated with inflammatory processes and oxidative stress [10–12]. They also have been related to genotoxic and cytotoxic effects [13]. Even though these studies have been carried to determine the toxicity of microplastics, none have reached the conclusive results necessary to definitively characterize these materials as either harmful or toxic [14]. This is mainly due to the difficulty of assessing the interactions of microplastics and nanoplastics with biomolecules, cells, tissues, and organs. Consequently, new perspectives are required to determine both the chemical reactivity of these contaminants and their interactions with the environment.

Along with all the investigations about the consequences of the presence of micro- and nanoplastics in the environment, it was reported that nanoplastics may interact with other pollutants, such as antibiotics [15,16] Antibiotics are important drugs in modern medicine, but their mass consumption and poor waste management lead to pollution problems [17,18]. Micro- and nanoplastics come into contact with antibiotics [19], either in the environment as pollutants or within the human body, where micro- and nanoplastics have also been identified within organs [20]. The presence of micro- and nanoplastics in the environment has been reported to alter the bioaccumulation of pharmaceuticals such as antibiotics. Some reports indicate that the bioaccumulation factor increased 10-fold in the presence of microplastics [21]. This was named the *Trojan horse* effect [22]. Micro- and nanoplastics can modify the bioaccumulation of antibiotics in aquatic organism, and this could represent a toxicological risk.

To determine the potential risk of nanoplastics, the first step is to investigate the chemical reactivity of these compounds. Despite all the results reported until now, little is known about the interaction and therefore the chemical reactivity of nanoplastics. For this reason, the main idea of this quantum chemical investigation is to analyze the chemical reactivity of different oligomers of polylactic acid (PLA) and polystyrene (PS). Oligomers have been successfully used as models of polymers [23–25]. The common definition for nanoplastics is any plastic whose length is smaller than 100 nm; according to IUPAC, an oligomer molecule is *“a molecule of intermediate relative molecular mass, the structure of which essentially comprises a small plurality of units derived, actually or conceptually, from molecules of lower relative molecular mass”* [26]. In general, we can say that oligomers are contained within the definition of nanoplastics, although the term also applies for non-plastic polymers. The chemical reactivity of these oligomers is investigated through their capacity to either donate or accept electrons [25,27] and therefore, their capacity to oxidize other molecules. The interactions of PS and PLA with two antibiotics, metronidazole and ciprofloxacin, is also investigated. Both antibiotics are included in the World Health Organization’s list of essential medicines [28]. Their chemical structure incorporates electronegative elements such as nitrogen, oxygen and fluoride, which could play a role in their interactions with micro and nanoplastics. Furthermore, experimental studies have been conducted evaluating their interactions with various microplastics, including PS [15,29,30]. The hypothesis here is that oligomer may interact strongly with antibiotics, and this could be the first step to understand the chemical reactivity of nanoplastics and the *Trojan horse* effect. Once the antibiotics are bonded to nanoplastics, the capacity to donate or accept electrons is modified and therefore the potential reactivity. A possible interpretation of the *Trojan horse* effect can be explained with the results presented here.

## Computational details

Gaussian 16 [31] was used for all electronic calculations. Different initial conformations were evaluated, and it was observed that linear oligomers with alternating benzene rings (for PS and its derivatives) were the most stable. These linear oligomer conformations are a good representation of the polymer chain. Different approximations of the antibiotics to the oligomer were also tested. The structures described here are the most stable.

Geometry optimizations were carried out at a M06-2X/6–311 + G(2d,p) level of theory without symmetry restrictions [32–34]. Grimme´s D3 dispersion correction was applied to consider nonlocal long range van der Waals interactions [35]. Harmonic analyses verified local minima.

To determine the electron transfer process, electrodonator and electroacceptor powers defined by Gázquez *et al.* [36,37] were calculated as follows:


$$\omega -=(3I+A{)}^{2}/16(I-A)$$ (1)

$$\omega +=(I+3A{)}^{2}/16(I-A)$$ (2)

I and A are the vertical ionization energy and vertical electron affinity, respectively. I and A are defined as follows:


$$Y\to Y^{1+}+1e^{-}I=E(Y^{1+})-E(Y)$$ (3)

$$Y^{1-}\to Y+1e^{-}A=E(Y)-E(Y^{1-})$$ (4)

**Fig 1:**
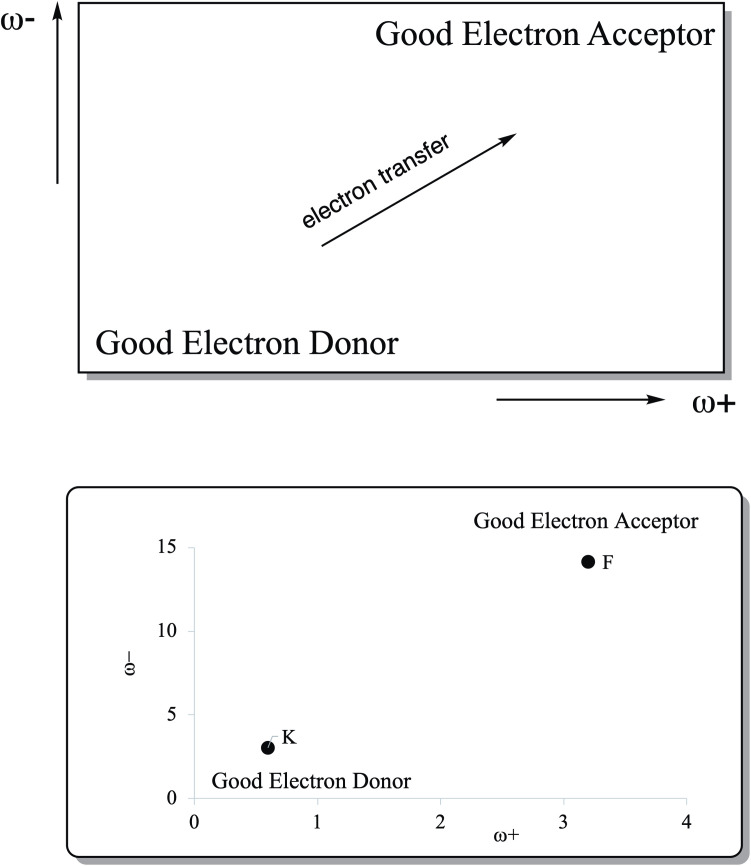
Electron Donor-Acceptor Map (DAM). F (good electron acceptor) and K (good electron donor) atoms are presented as reference. All values in eV.

Good electron acceptors will have large ω+ values and good electron donors will present small ω- values. These parameters refer to partial electron transfer reactions. With these chemical parameters it is possible to obtain the Donor Acceptor Map (DAM) as shown in Fig 1 [38]. In this map, the systems located down to the left are good electron donors whilst the systems located up to the right are good electron acceptors. Electrons will be transferred from atoms or molecules located down to the left to those located up to the right.

To investigate the stability of the interactions between oligomers and antibiotics, the interaction energy was obtained as follows:


$$E_{INT}=E\left(P\cdot \cdot \cdot Ant\right)-[E(P)+E(Ant)]$$ (5)

E(P**···**Ant) is the energy of the combined system. E(P) and E(Ant) are the electronic energy of the oligomer and the antibiotics, respectively. E_INT_ has a negative value when the energy of the combined system is smaller, and thus more stable, than the energy of the separated systems. BSSE is accounted for by incorporating the counterpoise correction [39,40]. Previous theoretical studies suggest the importance of considering the energy involved in the conformational change of the constituent molecules of a system to avoid overestimating the apparent stability of the complex [41]. The complexation and relaxation energies were calculated. They are reported in S1 Table of the Supporting Information. The relaxation energy associated with the change in molecular structure does not alter the conclusions about adduct formation in any of the systems presented in this work.

## Results and discussion

**Fig 2:**
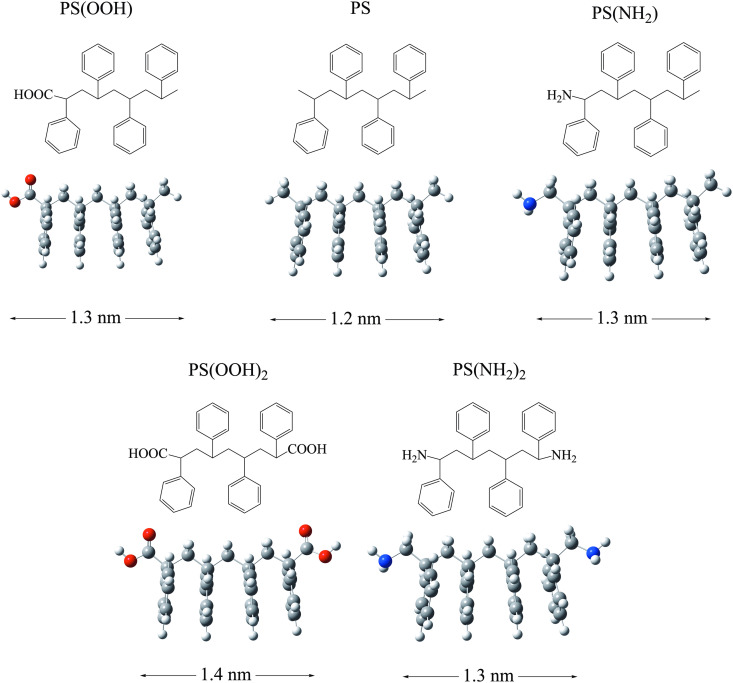
Molecular structures and optimized geometries of the oligomers of PS. The length of the molecules (in nm) is considered from one H atom to the H atom located on the opposite side of the molecule. Blue spheres represent nitrogen atoms. Red spheres represent oxygen atoms. Grey spheres represent carbon atoms. White spheres represent hydrogen atoms.

**Fig 3:**
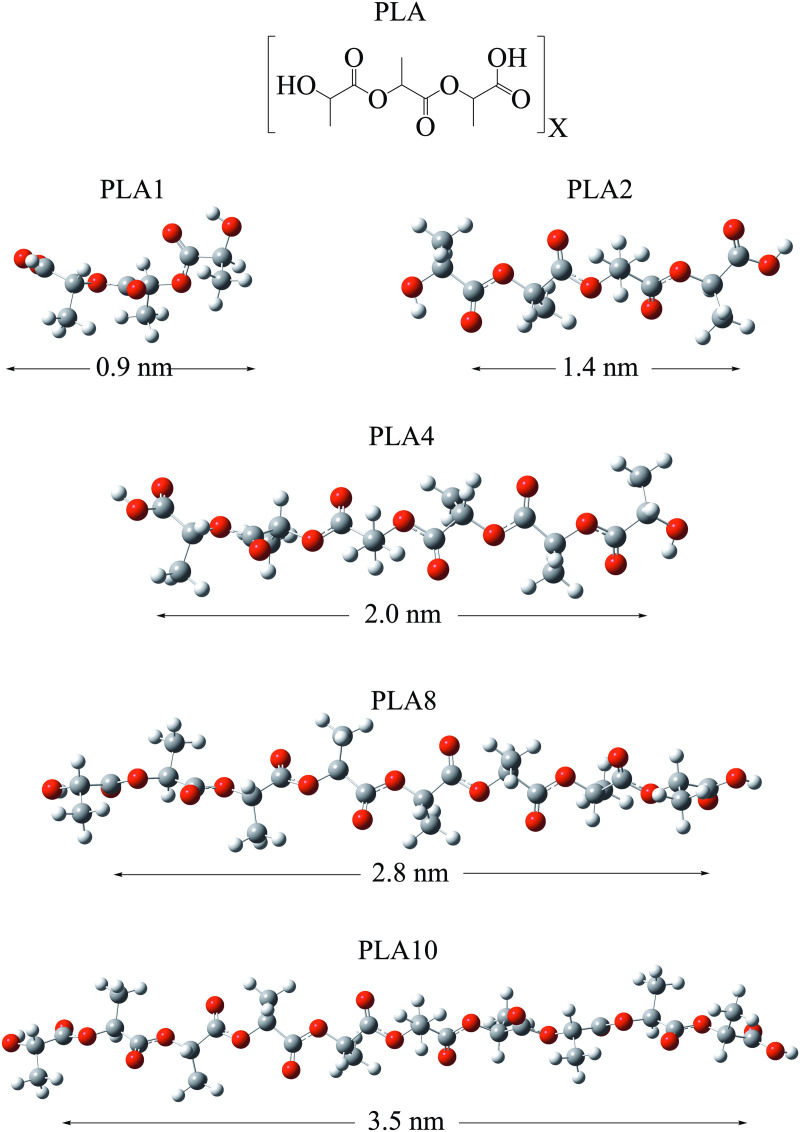
Molecular structures and optimized geometries of the oligomers of PLA. The length of the molecules (in nm) is considered from one H atom to the H atom located on the opposite side of the molecule. Red spheres represent oxygen atoms. Grey spheres represent carbon atoms. White spheres represent hydrogen atoms.

**Fig 4:**
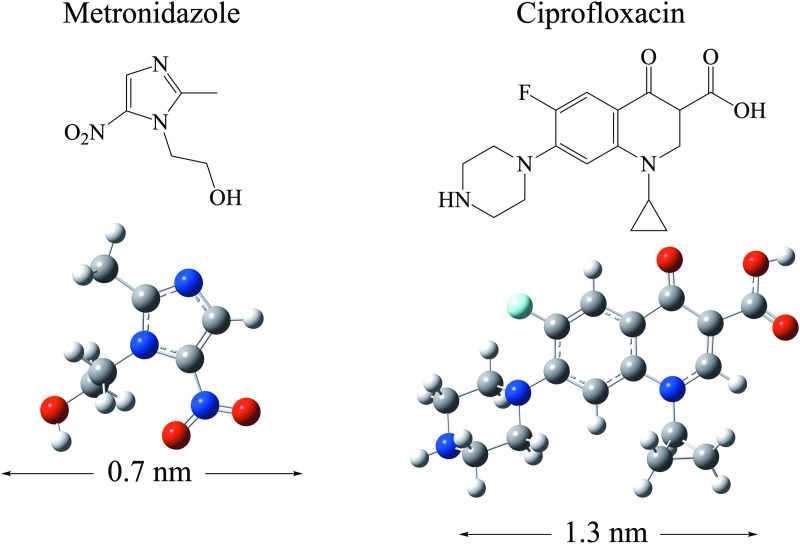
Molecular structures and optimized geometries of metronidazole and ciprofloxacin. The length of the molecules (in nm) is considered from one H atom to the H atom located on the opposite side of the molecule. Blue spheres represent nitrogen atoms. Red spheres represent oxygen atoms. Grey spheres represent carbon atoms. White spheres represent hydrogen atoms. Light blue sphere represents fluorine atom.

The optimized structures of all the oligomers and the antibiotics (metronidazole and ciprofloxacin) are shown from Figs 2–4. In this work, the influence of different elements in the chemical reactivity of oligomers was considered through the functionalized oligomers of PS, with either amino (NH_2_) or carboxyl groups (COOH). One or two amino and carboxyl groups were located at the ends of the carbon chain. We also investigated systems with different number of carbon atoms to form PLA oligomers, ranging from the monomer to the decamer (ten monomers). Metronidazole and ciprofloxacin are commonly used antibiotics, with metronidazole (0.7 nm) being half the size of ciprofloxacin (1.3 nm). Ciprofloxacin has one fluorine atom.

PS oligomers are linear, and all have a main carbon chain of nine atoms. The benzene groups do not overlap, as they alternate to avoid steric hindrance. Despite the functional groups, the length of all PS oligomers is similar, since they all have a main chain of nine carbon atoms. PLA oligomers are linear. In these systems, the length of the molecules is different since the main carbon chain is not the same in all cases. With the PLA oligomers, we can observe the effect of molecular length on chemical properties. The C-O bond length (1.5 Å) and the C-C bond distance (1.2 Å) are similar in all systems. All PLA oligomers have one COOH group and one COH group at the end of the chain, just like the monomer.

**Fig 5:**
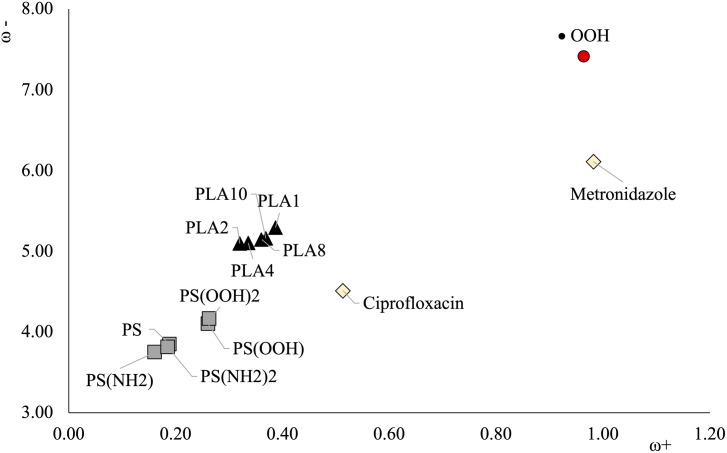
DAM of different oligomers and antibiotics. •OOH is included for comparison. Values are in eV.

DAM for all compounds under study is shown in Fig 5. Values for the free radical (•OOH) are included for comparison. Table 1 reports the ionization energies (I), electron affinities (A), and the values of the electron donor and acceptor powers (ω- and ω +) for all the compounds under study. It is important to note that all oligomers have negative values of the electron affinity, which means that they are not good electron acceptors. The values of ω + are also small. The conclusion that emerges from these values is that these oligomers are weak electron acceptors. This is not the case for the antibiotics and •OOH, where the electron affinity for these systems is positive, thus showing that these molecules are able to accept electrons (ciprofloxacin is the worse electron acceptor among these molecules). This is also observed in the ω + values. Although these values of ω + are small, we can use them to compare systems and analyze the electron transfer process.

**Table 1:** Ionization energies (I), electron affinities (A), and the values of the electron donor and acceptor powers (ω- and ω +) of all the systems under study. All values in eV.

	I	A	ω-	ω +
PS	8.32	−1.01	3.85	0.19
PSNH_2_	8.29	−1.12	3.74	0.16
PS(NH_2_)2	8.25	−1.00	3.81	0.19
PSOOH	8.43	−0.75	4.10	0.26
PS(OOH)2	8.56	−0.76	4.16	0.26
PLA1	10.53	−0.73	5.29	0.39
PLA2	10.49	−0.95	5.09	0.32
PLA4	10.40	−0.87	5.10	0.34
PLA8	10.34	−0.78	5.14	0.36
PLA10	10.32	−0.74	5.16	0.37
•OOH	12.51	0.39	7.42	0.96
Metronidazole	9.51	0.74	6.11	0.98
Ciprofloxacin	7.95	0.04	4.51	0.51

All oligomers under investigation have values of ω+ between 0.16 and 0.39 eV. Electron donor powers (ω-) values range from 3.74 to 5.29 eV. None of the PS oligomers are better electron acceptors than any of the PLA oligomers. It appears that the presence of oxygen atoms increases the capacity to accept electrons. PLA oligomers are better electron acceptors than PS oligomers, but PS oligomers are the best electron donors among all the studied compounds. Those with NH_2_ groups are better electron donors than the others. The size of the PLA oligomers does not have a great influence on the electron donor acceptor properties. All PLA oligomers present similar ω- and ω+ values. PLA1 is slightly better electron acceptor than the others. PLA2 and PLA10 are slightly better electron donors than other PLA oligomers.

PLA and PS oligomers are better electron donors but worse electron acceptors than •OOH. It is well known that this free radical is responsible of the oxidative stress. Because it is a good electron acceptor, this molecule accepts electrons from other molecules, which are then oxidized. This cannot be expected from the results of these oligomers. The capacity to accept electrons is not as good as the electron acceptor capacity of •OOH, as its values are lower by more than half. Therefore, it is not likely that these PS oligomers (or nanoplastics) could induce oxidative stress accepting electrons from other molecules. For PLA nanoplastics, according to our results, ω+ and ω- values are similar regardless the size of the system. Up to the decamer, it is possible to say that these oligomers are not as good electron acceptors as •OOH, and therefore, they will not induce oxidative stress accepting electrons from other molecules.

Analyzing the antibiotics, both are better electron acceptors than all the investigated oligomers. Ciprofloxacin is a better electron donor and worse electron acceptor than metronidazole. Metronidazole is as good an electron acceptor as •OOH and might oxidize other molecules. According to the DAM, electrons will be transferred from molecules located down to the left to those up to the right. This means that metronidazole will accept electrons from all oligomers.

**Fig 6:**
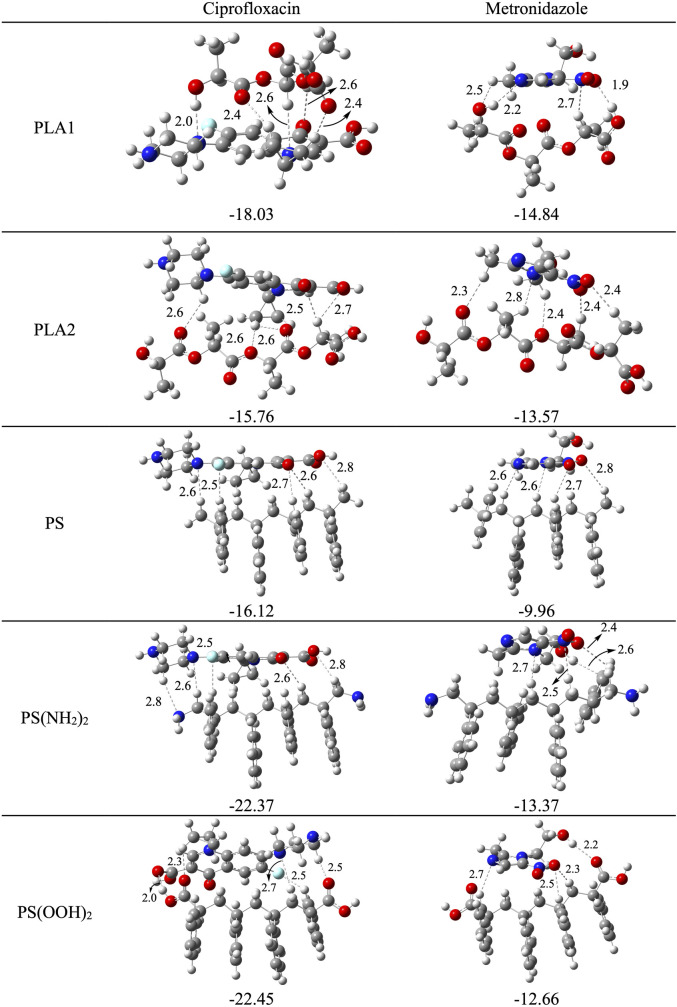
Optimized structures and interaction energies of the systems formed by oligomers with antibiotics. All values in kcal/mol. Hydrogen bond lengths are reported in Å. O in red, N in blue, F in light blue, C in grey, H in white.

The electron transfer process is important, but also the interaction energies with the antibiotics. In Fig 6 we report the interaction energies associated with different combined systems. We considered the PLA monomer (PLA1) and dimer (PLA2), as well as the oligomer of PS and the functionalized PS oligomers with two NH_2_ and two COOH groups. In Fig 6 we also report the optimized geometries. Different approximations of the antibiotics to the oligomer were considered, but in this table, we only report the most stable structures. As shown below, the interaction energies of ciprofloxacin with all oligomers are higher than those for metronidazole with all oligomers.

Analyzing the optimized structures, it is possible to find hydrogen bonds which contribute to the stabilization of the systems. The ciprofloxacin with PLA1 system has the third highest interaction energy, and there are five hydrogen bonds between ciprofloxacin and PLA1 in the structure. Metronidazole with PLA1 presents four hydrogen bonds, and the interaction energy is 1.2 times smaller than the interaction energy of PLA1 with ciprofloxacin. Comparing the interaction energies of antibiotics with PLA1 and PLA2, it is observed that the former has higher values than the latter. With ciprofloxacin, both present the same number of hydrogen bonds, but the hydrogen bond distances are shorter in PLA1-ciprofloxacin than in PLA2-ciprofloxacin. This is reflected in the E_int_ values and would suggest that longer oligomers tend to form less stable adducts than smaller ones. PS(OOH)_2_ with ciprofloxacin has five hydrogen bonds, and the interaction energy is the largest of all systems. PS(OOH)_2_ with metronidazole has four hydrogen bonds and the interaction energy is smaller than for PS(OOH)_2_ with ciprofloxacin. PS(NH_2_)_2_-ciprofloxacin has five hydrogen bonds. The interaction energy (−22.37 kcal/mol) is similar to the interaction energy of PS(OOH)_2_-ciprofloxacin (−22.45 kcal/mol), and both present similar hydrogen bonds. PS with metronidazole has the smallest interaction energy. The number of hydrogen bonds is four and the hydrogen bond lengths are larger in this system than in the others.

**Fig 7:**
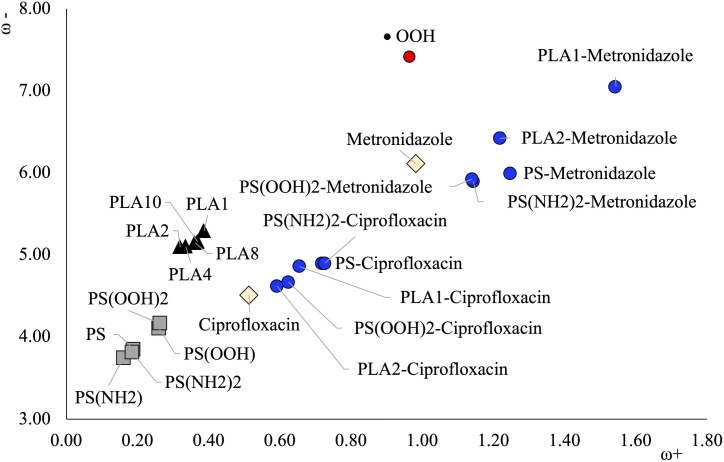
DAM of different oligomers and antibiotics and their composites. •OOH is included for comparison. Values are in eV.

The *Trojan horse* effect refers to the idea that the presence of nanoplastics increase the bioaccumulation of drugs such as antibiotics [22]. One explanation is that they interact with drugs and form stable compounds, as values of Fig 6 indicate. Oligomers could scavenge antibiotics such as metronidazole and ciprofloxacin from the environment; however, stable compounds formed by oligomers and antibiotics remain in the environment, increasing bioaccumulation. Previous research [19] has suggested that the interaction of microplastics with antibiotics could contribute to antibiotic resistance through the *Trojan Horse* effect, where the antibiotic-microplastic system gradually releases antibiotics expanding their range of pollution. Furthermore, the combined system could promote high concentration regions of antibiotics which incentivizes the proliferation of antibiotic-resistant bacteria [42]. Another possible consequence is that this interaction could interfere with the degradation of both oligomers and antibiotics. Oxidation reactions are one of the mechanisms for the degradation of antibiotics. It is possible that once antibiotics bind to nanoplastics or oligomers, oxidation becomes less viable. This can be analyzed with the electron transfer capacity. The electron donor acceptor powers for the combined systems of oligomers with antibiotics (those of Fig 6) were calculated. The correspondent DAM is reported in Fig 7 and the associated values are reported in Table 2. The electron donor acceptor capacity of the most combined systems is different from their constituent separated systems. All systems bonded to ciprofloxacin and metronidazole become better electron acceptors than the separated oligomers and antibiotics. Moreover, oligomers with metronidazole are better electron acceptors than •OOH, and this could contribute to the generation of reactive oxygen species.

**Table 2:** Ionization energies (I), electron affinities (A), and the values of the electron donor and acceptor powers (ω- and ω +) of all the systems under study. All values in eV.

	I	A	ω-	ω +
PS- Ciprofloxacin	7.91	0.45	4.90	0.72
PS(NH_2_)_2_-Ciprofloxacin	7.87	0.47	4.90	0.73
PS(OOH)_2_-Ciprofloxacin	7.79	0.29	4.67	0.62
PS- Metronidazole	8.29	1.20	5.99	1.25
PS(NH_2_)_2_-Metronidazole	8.45	1.06	5.90	1.14
PS(OOH)_2_-Metronidazole	8.52	1.05	5.92	1.14
PLA1-Ciprofloxacin	8.09	0.31	4.86	0.66
PLA2-Ciprofloxacin	7.84	0.22	4.62	0.59
PLA1-Metronidazole	9.49	1.51	7.04	1.54
PLA2-Metronidazole	9.30	1.11	6.43	1.22

The oxidation (electron loss) is related to the electron donor capacity. The systems formed with oligomers and ciprofloxacin present worse electron donor capacity (larger ω- values) than the isolated antibiotic. Therefore, the interaction of oligomers with ciprofloxacin reduces its oxidation capacity, making the degradation of this antibiotic via oxidation reactions less viable. With metronidazole, the electron donor capacity is similar to metronidazole for PS and its derivatives compounds, and the oxidation reactions are expected to be alike. The interaction with these oligomers should not affect the degradation process of metronidazole via oxidation reactions. PLA1-Metronidazole and PLA2-Metronidazole are worse electron donors than metronidazole. The interaction of PLA1 and PLA2 with metronidazole reduces the oxidation capacity of the antibiotic. This could interfere with its degradation process via oxidation reactions, increasing its bioaccumulation.

The results presented here show that the presence of oligomers could interfere with the degradation reactions of antibiotics by increasing their capacity to donate electrons, making their degradation more difficult in the presence of oxidizing species. This investigation gives us more information about the effect of nanoplastics on the environment and offer a possible explanation for the *Trojan horse* effect.

## Conclusions

The electron donor acceptor capacity of PLA and PS oligomers indicates that they are worse electron acceptors than ciprofloxacin and metronidazole. Ciprofloxacin is better electron donor and worse electron acceptor than metronidazole. Metronidazole is as good electron acceptor as •OOH and might oxidize other molecules. According to the DAM, electrons will be transferred from molecules located down to the left to those up to the right. This means that metronidazole could accept electrons from all oligomers.

None of the PS oligomers are better electron acceptors than any of the PLA oligomers. It appears that the presence of oxygen atoms increases the capacity to accept electrons. PS oligomers are the best electron donors among all the studied compounds and those with NH_2_ groups are better electron donors than the others. The length of the PLA oligomers is not related to the electron donor acceptor properties. All PLA oligomers present similar ω- and ω+ values. There is no overall trend regarding oligomer size and electron donor-acceptor capacity. For example, the smallest PLA oligomer (PLA1, the monomer) is the best electron acceptor, but PLA8 is a better electron acceptor than PLA2.

The interaction of antibiotics and oligomers is through hydrogen bonds, where the number and bond length of these hydrogen bonds determines the interaction energy. The interaction energies of ciprofloxacin with all oligomers are higher than those for metronidazole with all oligomers. There are more, and shorter, hydrogen bonds in the systems with ciprofloxacin. PS(OOH)_2_-ciprofloxacin is the most stable compound followed by PS(NH_2_)_2_-ciprofloxacin and PLA1- ciprofloxacin.

Considering that oxidation is related to the electron donor capacity, the systems formed with oligomers and ciprofloxacin present worse electron donor capacity (larger ω- values) than the isolated antibiotic. PLA1- metronidazole and PLA2-metronidazole are also worse electron donors than metronidazole; therefore, the interaction of these oligomers with ciprofloxacin and metronidazole might hinder the oxidation reactions and therefore the degradation of these antibiotics. For metronidazole with PS and its derivatives, the electron donor capacity is similar to the value of isolated antibiotic, and the oxidation reactions are expected to be alike; as a result, the interaction with these oligomers should not affect their degradation process. All systems with oligomers and metronidazole are better electron acceptors than metronidazole. All complex systems present different electron donor-acceptor capacities than their respective isolated antibiotic, which could inhibit their effectiveness against certain bacteria.

The formation of stable compounds between oligomers and antibiotics helps explain the *Trojan Horse* effect, where organic pollutants, such as antibiotics, can be sequestered by nanoplastics, thereby preventing their oxidation and degradation. Since they do not degrade, they can bioaccumulate, which explains the so-called *Trojan Horse* effect. Although this work is focused on the study of oligomer-antibiotic compounds, we consider that the interactions within these systems could also be present in the microplastic-antibiotic systems. Further studies are needed to understand the reaction mechanism through which the adducts are formed, as well as to determine the interactions between antibiotics and larger polymer systems.

## Supporting information

S1 Table Interaction energy, complexation energy, and relaxation energy of all oligomer and antibiotic adduct systems under consideration. All values are reported in kcal/mol.
(PDF)
