# Supplementary material for: Interaction of nanoplastics with metronidazole and ciprofloxacin: The Trojan Horse effect
Source: PLoS One. 2025 Aug 20;20(8):e0330708. doi: 10.1371/journal.pone.0330708 (PMC12367159; doi:10.1371/journal.pone.0330708)
Supplement: S1 Table — All values are reported in kcal/mol. (PDF) [file pone.0330708.s001.pdf]

S1 Table. Interaction energy, complexation energy, and relaxation energy of all oligomer and antibiotic adduct systems under consideration. All values are reported in kcal/mol.

	Interaction Energy	Complexation Energy	Relaxation Energy
PLA1-Ciprofloxacin	-18.03	-22.39	4.37
PLA1-Metronidazole	-14.84	-18.11	3.27
PLA2-Ciprofloxacin	-15.76	-18.80	3.04
PLA2-Metronidazole	-13.57	-12.51	-1.06
PS-Ciprofloxacin	-16.12	-17.25	1.13
PS-Metronidazole	-9.96	-10.67	0.71
PS(NH)2-Ciprofloxacin	-22.37	-19.50	-2.86
PS(NH)2-Metronidazole	-13.37	-15.07	1.70
PS(OOH)2-Ciprofloxacin	-22.45	-26.56	4.10
PS(OOH)2-Metronidazole	-12.66	-15.89	3.22
